# Photogrammetry in Spinal Assessment: A Comparative Analysis with Traditional Clinical Methods

**DOI:** 10.3390/jcm14124032

**Published:** 2025-06-06

**Authors:** Nicolae-Adrian Jurjiu, Ciprian Glazer, Mihaela Oravitan, Corina Pantea, Claudiu Avram

**Affiliations:** 1 Faculty of Physical Education and Sport, University of West Timișoara, 300223 Timișoara, Romania; nicolae.jurjiu@e-uvt.ro (N.-A.J.); mihaela.oravitan@e-uvt.ro (M.O.); corina.pantea@e-uvt.ro (C.P.); 2 Faculty of Medicine, “Victor Babeș” University of Medicine and Pharmacy, 300041 Timișoara, Romania; claudiu.avram@gmail.com

**Keywords:** spinal mobility, photogrammetry, clinical rehabilitation

## Abstract

**Background/Objectives**: Spine mobility is essential for overall health and daily functioning. Accurate assessment of spinal mobility is necessary for diagnosing and managing orthopedic, neurological, and rheumatological disorders, particularly in patients experiencing lower back and thoracic pain. The present study evaluates the effectiveness of traditional clinical tests in comparison to the innovative photogrammetric 3D posture assessment for evaluating spinal mobility. **Methods**: A total of 20 patients from a medical clinic underwent tests to measure lumbar and thoracic spine mobility, including flexion, lateral incline, and axial rotation, using both conventional and 3D posture assessment methods. **Results**: We found strong correlations between investigated methods, which recommends photogrammetry as a reliable and effective tool for assessing posture in clinical practice. Furthermore, 3D posture assessment offers a faster approach (clinical evaluation: 2:59 ± 0.22 min vs. photogrammetry: 1:03 ± 0.01 min) and a more practical method for assessing spinal mobility, thereby enhancing the patient experience and providing clinicians with objective data for treatment planning. **Conclusions**: The study highlights the value of modern technologies in clinical assessment and therapeutic intervention, encouraging therapists to integrate photogrammetric methods into their daily practice to improve patient outcomes.

## 1. Introduction

The spine’s mobility is important for the optimal functioning of the human body, as it affects both musculoskeletal functioning and overall health. An accurate evaluation of spinal mobility is essential for diagnosing and managing orthopedic, neurologic, and rheumatologic disorders in clinical practice.

Assessing spinal mobility is a critical clinical process, particularly for patients experiencing lower back and thoracic pain. This clinical step is important for obtaining an accurate diagnosis and designing effective rehabilitation plans [1]. Another aspect of assessing spinal mobility is the correlation between the results and the overall health status of patients. Regional pain is quite common and can come from various causes, like degenerative conditions, injuries, or muscular and skeletal problems. Sometimes, this pain becomes chronic and is often connected to issues like anxiety and depression, which can affect how we feel the pain and how well treatments work [2].

Conventional spinal mobility is assessed using standardized clinical tests that measure the range of motion in multiple planes, providing valuable insights into patients’ functional status [3].

Potapenco R. indicates that lower back pain ranks among the most prevalent health concerns within the population, substantially affecting the quality of life of people [1]. Assessment of spinal mobility is useful in identifying the etiological factors of pain, as well as in determining its severity and the consequences for the patient’s daily activities. Assessment methodologies may encompass tests such as the Finger-to-Floor Index, which evaluates lumbar flexion and thoracic rotation, which are critical for diagnosing the functional limitations experienced by patients [3]. These clinical evaluations are used further in developing an appropriate treatment strategy, which may incorporate physical therapy, pharmacological management, or surgical options, depending on the severity of the clinical condition.

While traditional clinical tests like the Finger-to-Floor Index and thoracic rotation remain essential for assessing spinal mobility and guiding treatment, recent technological advances have introduced new ways to evaluate posture and movement. Modern methods such as photogrammetry and 3D motion capture, originally used in gaming and engineering, were recently adapted for medical use. These techniques allow real-time, accurate body measurements in three dimensions, offering a non-invasive, detailed view that complements traditional assessments.

Photogrammetry is an assessment method based on analyzing images captured from different angles, which allows for the precise recreation and measurement of objects or shapes in three-dimensional space. This technique involves capturing digital images and using them to create accurate 3D models of the studied area or object. In our context, photogrammetry enables a detailed and precise evaluation of posture or movement, providing a non-invasive and efficient alternative for clinical measurements.

The 3D posture assessment has been adapted for medical applications and has proven effective in assessing posture and mobility in patients with various musculoskeletal conditions. This technology enables the real-time capture of movements, providing physical therapists with valuable insights into patients’ functional limitations [4]. By analyzing the acquired data, physical therapists can identify abnormal movement patterns that may contribute to lower back or thoracic pain, thereby facilitating more targeted therapeutic interventions [5].

Contemporary methods that employ 3D motion capture technology aim to enhance the accuracy of spinal movement analysis by providing a more comprehensive assessment of these movements. Research indicates that devices such as the Microsoft Kinect can quantify spinal motion across multiple dimensions, capturing real-time data with a high degree of spatial precision [6,7]. The heightened accuracy of 3D systems facilitates a deeper understanding of spinal mechanics, including critical parameters such as angular velocity and acceleration, which traditional techniques frequently fail to capture adequately [8].

Nevertheless, despite the advanced capabilities of 3D assessment technologies, they are not without their challenges. Integrating these systems into clinical practice is often limited by the complexity of the setup and the technical expertise required for effective implementation. Additionally, concerns have been raised regarding the cost-effectiveness and user-friendliness of these sophisticated technologies in everyday clinical scenarios, particularly in resource-constrained environments. While systems like Kinect were initially designed to provide low-cost and accessible alternatives, their practicality continues to spark debate [9,10].

The strength of the data obtained from 3D motion capture systems can potentially transform clinical practices significantly. For instance, a recent study utilizing Kinect technology demonstrated its effectiveness in detecting subtle variations in spinal alignment that might be overlooked by conventional methods [11]. Furthermore, these technologies hold promise in predictive analytics, such as tracking disease progression in patients with axial spondyloarthritis, which can significantly influence treatment pathways and rehabilitation strategies [12]. Such insights support a more interactive approach to patient care, promoting a patient-centered perspective that adapts to individual needs based on detailed measurements.

The ongoing advancements in human motion capture using 3D imaging are set to bridge the gap between traditional assessment methods and the future of clinical evaluation. Recent developments have improved the accuracy of spinal alignment measurements and introduced automated tools to aid in the clinical decision-making process [8,13]. These dimensions of technological intervention reflect a broader trend within the field of medicine that emphasizes the increasing importance of data analytics and personalized treatment plans [10].

Moreover, the 3D posture assessment method can be used for assessments and therapeutic interventions. Exercise programs that utilize this device’s visual feedback can help patients enhance their mobility, alleviate pain, and help them become aware of their posture in real time [14].

This article compares two distinct methods of evaluating spinal mobility: the conventional method, which relies on traditional clinical tests, and an innovative approach that utilizes 3D posture assessment techniques. By systematically examining the advantages and disadvantages of each method, this study seeks to provide meaningful insights into the efficacy and relevance of 3D posture assessment in clinical practice.

The article aims to explore how integrating 3D technologies can enhance the accuracy and detail of spinal mobility evaluations, compared to conventional methods that may lack precision or adaptability in capturing dynamic postural changes. The study intends to highlight the potential of 3D photogrammetry for detecting subtle deviations in spinal alignment and mobility, which can help guide therapeutic interventions.

## 2. Materials and Methods

We designed a comparative study to evaluate two distinct methods for assessing spinal mobility: traditional clinical evaluations and the innovative application of 3D photogrammetric posture assessment. This investigation seeks to elucidate the strengths and weaknesses inherent in each approach, providing a comprehensive understanding of their respective impacts on clinical practice.

Over three months, we evaluated 62 patients with thoracic and low back pain at a local rehabilitation clinic for potential study participation. After a consultation with the rehabilitation doctor, participants were diagnosed with lumbago and/or thoracic back pain, which constituted our inclusion criterion. The exclusion criteria included back pain from non-musculoskeletal conditions and various congenital or inherited skeletal deformities. Ultimately, twenty individuals provided informed consent to take part in this study, with an average age of 32.7 ± 4.31 years.

Subjects underwent clinical assessment tests of lumbar and thoracic spine mobility when flexion, lateral incline, and rotation movements of the spine were recorded. We used the “fingers to the ground” test for flexion and lateral incline movement; the methodology was adopted after a study conducted by Perret C., in which the reliability of this test was demonstrated in patients with back pain [15]. The subject stands barefoot with their feet positioned close together, ensuring that their weight is evenly distributed between both feet. It is essential to perform the test on a flat, stable surface to prevent any slips or falls. As the patient reaches down, they should keep their legs straight and avoid bending their knees. This movement should be executed slowly and with control. We will then measure the distance from the tips of their fingers to the ground, recording this measurement in centimeters.

To evaluate the trunk rotation, the subject is sitting and instructed to rotate the trunk to the left and right as far as possible, maintaining a stable pelvic position and avoiding compensatory movements such as lateral inclination or hip rotation. The extent of rotation is measured using a metric tape and recorded in centimeters.

Posture assessments were also performed using a 3D infrared camera (Kinect Azure, Microsoft, Redmond, WA, USA), which can recognize 32 human body joints. The sensor assigns a three-dimensional value for each joint, representing its position in Cartesian space: the X coordinate represents the horizontal space, the Y coordinate represents the vertical space, and the Z coordinate represents the depth space. Using these values, the dedicated software can create a digital model.

**Figure 1:**
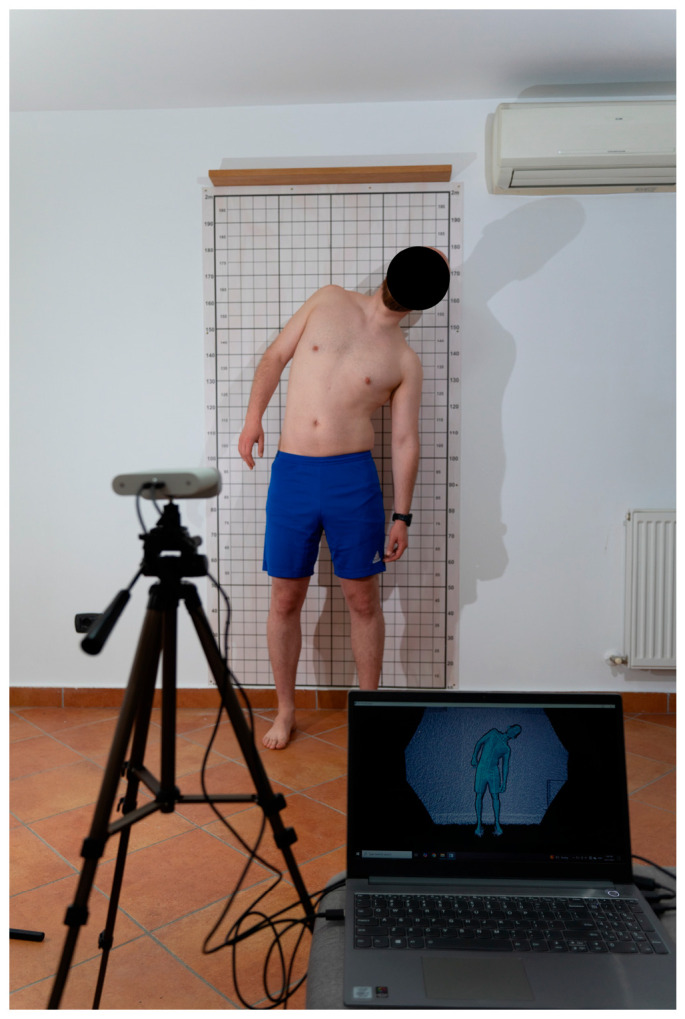
Example of photogrammetry.

We followed the recommendation provided by Microsoft to obtain accurate measurements using Kinect Azure. The patient was positioned at 2 m from the sensor, allowing precise movement capture. The sensor was placed at the patient’s hip level. The patient was barefoot and wore fitted clothing, such as a sports shirt and shorts (Figure 1).

With the support of a software engineer, we utilized the open-source software that controls the Kinect Azure sensor to extract these measurements, allowing us to calculate them automatically without the need for body stickers or other distinctive markers. To assess the mobility of flexion and lateral incline movements, the software calculates the distance between the tips of the fingers and the ground at the maximum amplitude point. This involved measuring the difference between the Y-coordinate value at the tips of the fingers and the Y-coordinate value at the level of the foot resting on the ground. We measured the difference between the Z-coordinate value at the shoulder level in the initial position and the value at the maximum amplitude point for trunk rotation movements.

All postural assessments were conducted by the same physical therapist following a diagnostic evaluation by the rehabilitation physician. Both assessments employing traditional and 3D photogrammetric techniques were performed within a short timeframe, one succeeding the other, to ensure methodological consistency and mitigate variability.

The statistical analysis was conducted by a different person from the one who performed the patient assessments, ensuring blinding to the assessment method used. This measure minimized potential bias and enhanced the objectivity of the data interpretation.

Statistical analysis was performed using GraphPad Prism version 10. Student’s *t*-test was utilized to evaluate the significance of differences between datasets. Additionally, Pearson’s correlation coefficient was used to assess the strength and direction of the relationships between clinical and photogrammetric measurements. To assess reliability or agreement, we used the following statistical measurements: Intraclass Correlation Coefficient (ICC), Standard Error of Measurement (SEM), Minimal Detectable Change (MDC), and Bland–Altman analysis.

## 3. Results

We performed a comprehensive evaluation of patients using both assessment methods. We organized the resulting data into a detailed table to aid our analysis. This method enabled us to identify the differences in spinal mobility assessments for all trunk movements. By presenting the data in this format, we aimed to provide an understandable comparison highlighting each method’s strengths and limitations, ultimately enhancing our understanding of how these techniques assess spinal mobility (Table 1).

The average evaluation time for the clinical assessments was recorded at 2 min and 59.3 s (±22 s), whereas the mean duration for the 3D posture assessment was 1 min and 2.5 s (±1 s).

When patients were asked to provide feedback on the evaluation methods, most participants (9 patients, 45%) preferred the photogrammetric method. In contrast, 6 patients (30%) reported no specific preference, while 5 patients (25%) favored the traditional assessment method. This feedback highlights the participants’ overall positive perception and acceptance of the photogrammetric approach.

**Figure 2:**
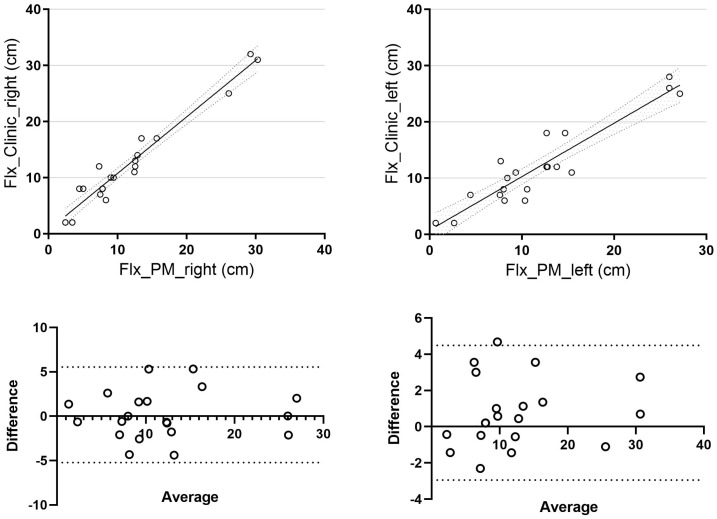
Trunk flexion correlation and Bland–Altman of flexion (difference vs. average) between clinical and photogrammetric methods. Flx_Clinic_right: right side trunk flexion assessment using the clinical method; Flx_Clinic_left: left side trunk flexion assessment using the clinical method; Flx_PM_right: right side trunk flexion assessment using the photogrammetric method; Flx_PM_left: left side trunk flexion assessment using the photogrammetric method.

A mean difference of 1.54 ± 1.28 cm was observed for trunk flexion on the right side and 2.16 ± 1.59 cm on the left side. When comparing clinical and photogrammetric methods, we found a strong and statistically significant correlation for the right side (r = 0.97, *p* = 0.04, 95% CI [0.93, 0.99]) and a correlation for the left side (r = 0.93), although it was not statistically significant (*p* = 0.39, 95% CI [0.83, 0.97]) (Figure 2).

**Figure 3:**
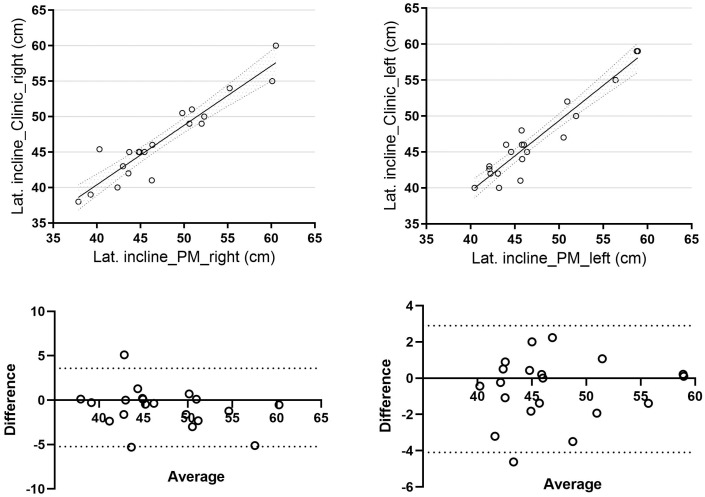
Lateral trunk inclination correlation Bland–Altman of lateral inclination (difference vs. average) between clinical and photogrammetric methods. Lat. incline_Clinic_right: right side trunk inclination assessment using the clinical method; Lat. incline_Clinic_left: left side trunk inclination assessment using the clinical method; Lat. incline_PM_right: right side trunk inclination assessment using the photogrammetric method; Lat. incline_PM_left: left side trunk inclination assessment using the photogrammetric method.

Regarding lateral trunk inclination, the mean difference between the two methods was 1.59 ± 1.72 cm for right-sided inclination and 1.36 ± 1.23 cm for left-sided inclination. A direct correlation was observed between the two methods (r = 0.94, *p* = 0.06, 95% CI [0.84, 0.97] for right-side inclination and r = 0.95, *p* = 0.07, 95% CI [0.88, 0.98] for left-side inclination), with results approaching statistical significance (Figure 3).

**Figure 4:**
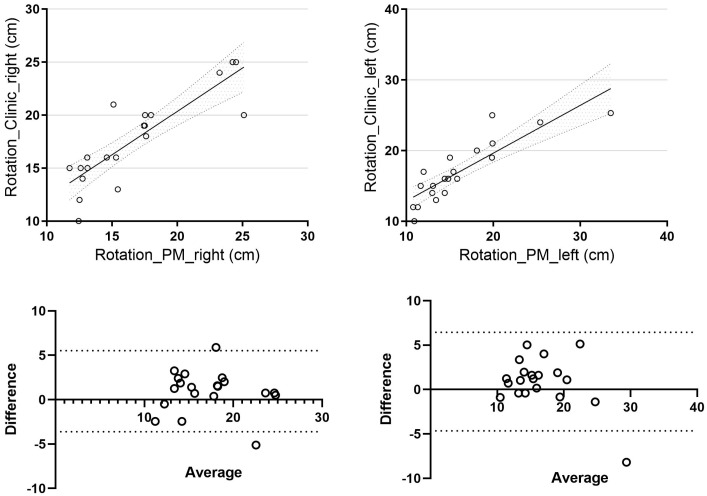
Trunk rotation correlation Bland–Altman of trunk rotation (difference vs. average) between clinical and photogrammetric methods. Rotation_Clinic_right: right side trunk rotation assessment using the clinical method; Rotation_Clinic_left: left side trunk rotation assessment using the clinical method; Rotation_PM_right: right side trunk rotation assessment using the photogrammetric method; Rotation_PM_left: left side trunk rotation assessment using the photogrammetric method.

For trunk rotation, the mean difference for right-sided rotation was 2.01 ± 1.43 cm, while for left-sided rotation, it was 2.11 ± 1.99 cm. When comparing the clinical and photogrammetric methods, we observed a strong correlation for right-sided rotation (r = 0.85, *p* = 0.04, 95% CI [0.65, 0.94]) and a correlation approaching statistical significance for left-sided rotation (r = 0.86, *p* = 0.08, 95% CI [0.68, 0.95]) (Figure 4).

## 4. Discussion

The study’s results highlight the differences in trunk posture assessments across various movements (trunk flexion, lateral inclination, and rotation). With a mean difference of 1.54 cm for trunk flexion on the right and 2.16 cm on the left, the results indicate that both assessments are comparable, reinforcing that the measurements are reliable and consistent. From a clinical point of view, we do not consider a difference of less than 3 cm meaningful, and it may not impact clinical decision-making.

For the evaluation of lateral trunk inclination, the mean differences of 1.59 cm for right-sided inclination and 1.36 cm for left-sided inclination suggest that the assessment methods yield similar results. Nonetheless, comparing both methods, we did not reach the statistical significance for right-sided and left-sided inclination, highlighting potential biomechanical or procedural factors that could influence measurements.

The correlations observed between the two assessment methods highlight the validity of 3D posture assessment as an effective clinical evaluation tool. These findings support the conclusions of previous studies, demonstrating that advanced technology can deliver accurate and reliable measurements comparable to traditional methods.

Furthermore, the statistical significance of the observed differences, particularly with *p*-values below 0.05 for right-sided flexion and rotation, suggests that these differences are unlikely to occur by chance and supports the study’s findings. The *p*-values between 0.05 and 0.10 for left-sided lateral inclination and rotation suggest that while these results approach significance, further research may be necessary to strengthen the conclusions drawn.

Some observed differences are statistically significant (right-sided flexion and left-right rotation), as indicated by *p*-values below 0.05. This suggests that these differences are unlikely to occur by chance and supports the study’s findings. On the other hand, the *p*-values for left-sided lateral inclination and rotation, which fall between 0.05 and 0.10, indicate that while these results are close to being significant, additional research may be needed to confirm these findings.

The clinical evaluation time averages approximately 2 min and 59 s, with a standard deviation of 22 s. This variability suggests that the time taken to complete clinical evaluations can range from about 2 min and 37 s to 3 min and 21 s. In contrast, the photogrammetric evaluation time is shorter, averaging around 1 min and 2.5 s, with a very small standard deviation of just 1 s. This indicates that the photogrammetric method is much more consistent across evaluations.

The results indicate that the photogrammetric method is effective for assessing mobility, offering a faster and more efficient approach. Another advantage of this method is that it does not require markers to be applied to the body for posture assessment. This may reduce examination time and enhance the patient experience, allowing clinicians to obtain objective information for treatment planning.

Using the photogrammetric method, joint mobility assessment leverages advanced depth-sensing and skeletal tracking technology to quantitatively evaluate dynamic movement parameters. Studies have demonstrated that the photogrammetric method delivers highly reliable and repeatable measures of motor function across various domains—from dynamic knee measurements [16] and temporal gait parameters [17] to complex tasks such as the sit-to-stand test [18] and the Timed Up and Go (TUG) test [19]. Improved hardware and enhanced motion-tracking algorithms support its application in clinical settings, where subtle changes in joint mobility can improve therapeutic decisions [20,21].

It is essential to recognize the limitations of this study, particularly the potential impact of individual variability on the results, including factors such as age, gender, body mass index (BMI), and the patients’ medical history. Furthermore, the relatively small sample size and focus on patients with spinal conditions may limit the generalizability of the findings. Conducting additional studies with a more diverse and larger sample will be crucial to validate these observations and enhance the applicability of the results. Another limitation of the study is that we did not use a gold standard motion analysis system (e.g., optical 3D marker-based system) for the comparison of postural assessment, primarily because it is not a standard facility available in medical clinics.

Future research could explore the effects of different types of interventions, such as therapeutic exercises that utilize photogrammetric feedback, on spinal mobility. This would enhance our understanding of the role of technology in rehabilitation. Additionally, conducting longitudinal observations of patients could provide valuable insights into the long-term evolution of their spinal mobility.

## 5. Conclusions

The photogrammetric method for assessing spinal mobility is comparable to the traditional clinical method in terms of reliability. It offers the benefits of a quick, objective, and noninvasive assessment. Although strong correlations were observed between the two methods, not all reached statistical significance. Despite this limitation, the use of this technology in clinical practice could be beneficial.

The study highlights the value of modern technologies in clinical assessment and therapeutic intervention, encouraging therapists to integrate photogrammetric methods into their daily practice to improve patient outcomes.

## Figures and Tables

**Table 1:** Mean data of spine mobility assessments using clinical and photogrammetric methods.

	Photogrammetry Measurements	Clinical Measurements	*p*	ICC	SEM	MDC
**Flx_right**	11.88 ± 7.83	12.65 ± 8.09	0.04	0.93	0.58	1.60
**Flx_left**	11.93 ± 7.11	12.10 ± 7.26	0.40	0.87	1.08	3.00
**Lat. incline_right**	47.47 ± 6.20	46.65 ± 5.55	0.06	0.78	1.04	2.87
**Lat. incline_left**	47.23 ± 5.41	46.63 ± 5.58	0.07	0.67	1.28	3.53
**Rotation_right**	16.69 ± 4.27	17.65 ± 4.09	0.04	0.42	2.16	5.99
**Rotation_left**	16.12 ± 5.42	17.02 ± 4.24	0.08	0.38	2.79	7.72

ICC: Intraclass Correlation Coefficients, SEM: Standard Error of Measurement, MDC: Minimal Detectable Change.

## Data Availability

The original contributions presented in this study are included in the article. Further inquiries can be directed to the corresponding author.
